# Causes of Anemia in Polish Older Population—Results from the PolSenior Study

**DOI:** 10.3390/cells10082167

**Published:** 2021-08-22

**Authors:** Arkadiusz Styszyński, Jerzy Chudek, Małgorzata Mossakowska, Krzysztof Lewandowski, Monika Puzianowska-Kuźnicka, Alicja Klich-Rączka, Andrzej Więcek, Katarzyna Wieczorowska-Tobis

**Affiliations:** 1 Laboratory for Geriatric Medicine, Department of Palliative Medicine, Poznan University of Medical Sciences, 61-245 Poznan, Poland; tobis@ump.edu.pl; 2 Department of Internal Medicine and Oncological Chemotherapy, Medical University of Silesia, 40-029 Katowice, Poland; chj@poczta.fm; 3 International Institute of Molecular and Cell Biology, 02-109 Warsaw, Poland; mmossakowska@iimcb.gov.pl; 4 Department of Laboratory Medicine, Medical University of Gdansk, 80-211 Gdansk, Poland; klewandowski@gumed.edu.pl; 5 Department of Human Epigenetics, Mossakowski Medical Research Institute, Polish Academy of Sciences, 02-106 Warsaw, Poland; mpuzianowska@imdik.pan.pl; 6 Department of Geriatrics and Gerontology, Medical Centre of Postgraduate Education, 01-813 Warsaw, Poland; 7 Department of Internal Medicine and Gerontology, Jagiellonian University, Collegium Medicum, 31-531 Krakow, Poland; ala_klich@o2.pl; 8 Department of Nephrology, Transplantation and Internal Medicine, Medical University of Silesia, 40-055 Katowice, Poland; awiecek@sum.edu.pl

**Keywords:** iron deficiency, folate deficiency, vitamin B_12_ deficiency, anemia of inflammation, chronic kidney disease, aging

## Abstract

Vitamin B_12_, folate, iron deficiency (IDA), chronic kidney disease (CKD), and anemia of inflammation (AI) are among the main causes of anemia in the elderly. WHO criteria of nutritional deficiencies neglect aging-related changes in absorption, metabolism, and utilization of nutrients. Age-specific criteria for the diagnosis of functional nutritional deficiency related to anemia are necessary. We examined the nationally representative sample of Polish seniors. Complete blood count, serum iron, ferritin, vitamin B_12_, folate, and renal parameters were assessed in 3452 (1632 women, 1820 men) participants aged above 64. Cut-off points for nutritional deficiencies were determined based on the WHO criteria (method-A), lower 2.5 percentile of the studied population (method-B), and receiver operating characteristic (ROC) analysis (method-C). Method-A leads to an overestimation of the prevalence of vitamin B_12_ and folate deficiency, while method-B to their underestimation with over 50% of unexplained anemia. Based on method-C, anemia was classified as nutritional in 55.9%. In 22.3% of cases, reasons for anemia remained unexplained, the other 21.8% were related to CKD or AI. Mild cases were less common in IDA, and more common in non-deficiency anemia. Serum folate had an insignificant impact on anemia. It is necessary to adopt the age-specific criteria for nutrient deficiency in an old population.

## 1. Introduction

According to World Population Prospects announced by United Nations in 2019, there were 703 million persons aged 65 years or over around the world. The number of older persons is projected to double by 2050 and the percentage will rise from 9% to 16%. Population aging concerning the number and proportion of older persons in the entire population is observed in every country in the world [1].

The demand for health care goods and services is linked to the age structure of the population and, notably, with the share of older people in the overall population. This is because older people often develop multi-morbidity conditions and have a higher probability of loss of independence, which requires costly medical and social care [2]. To prevent dependency affecting older people, it is crucial to put attention to common medical problems, especially those whose importance is not widely recognized. Anemia is one of these conditions. In the population of older Americans anemia was identified as an independent risk factor for higher hospitalization rate and more extended stay, as well as higher mortality [3]. Anemia in old age is associated with reduced muscle strength and physical performance, leading to the increased risk of falls and depression, as well as deterioration of cognitive functions [4,5,6]. Anemia is one of the leading causes of type 2 myocardial infarction in the geriatric population [7].

Proper therapeutic management aimed at eliminating anemia requires, first of all, its causes to be determined. The most common causes of anemia in older adults are nutritional deficiency, chronic kidney disease, chronic inflammation, and blood loss from the gastrointestinal (GI) tract resulting from GI pathology, although in many patients the etiology is unknown [8]. It is commonly believed that the most important nutritional factors in the pathogenesis of anemia are iron, folate, and vitamin B_12_ deficiency. The problem, however, arises when deciding what laboratory values for a nutrient are considered deficient in old age and whether the deficiency plays a significant role in the pathogenesis of anemia. It is also unclear whether factors considered to be the cause of anemia in young people are of equal importance in the etiology of this disorder in older age. For example, a study conducted by French investigators on the older adult population staying in long-term facilities showed that despite the folate deficiency, there was no deterioration of hematological parameters or the functional state [9].

It is, therefore, necessary to establish age-specific criteria for the diagnosis of nutritional deficiencies leading to anemia. The most common method of determining the reference value for a test is the lowest 2.5 percentile limit for a population of young healthy people. Based on this method, the WHO defines the following criteria for nutritional deficiencies: serum folate < 3 ng/mL or red blood cells (RBC) folate < 100 mg/mL, serum vitamin B_12_ < 200 pg/mL and (for iron deficiency) serum ferritin < 15 µg/mL or zinc protoporphyrin in RBC > 70–80 µg/dL [10,11,12,13]. However, adopting values established as 95% confidence interval of results of young healthy adults ignores the changes resulting from the physiologic aging process.

Therefore, some researchers postulate to define age-specific reference values for older adults. An example of such an approach is the study of researchers from Uppsala University, Sweden, who investigated 31 frequently used laboratory markers in over seven hundred 75-year-old persons without diabetes. In their opinion, the calculated lower 2.5 and upper 97.5 percentiles for these markers may serve as adequate reference values in the elderly population [14]. Although this approach takes into account age-related changes in biological markers, there is a dilemma whether the reference values should be based on the results obtained in a selected group of older adults in whom no significant pathology was found, or whether the reference group should represent a wide range of various variants of aging, including people with cardiovascular or kidney diseases, which are very common in the advanced age [15]. In the first case, the range of reference values will be narrow, which will cause that many clinically insignificant states of slightly impaired functioning of organs will be interpreted as a serious pathology. In the latter case, the range of reference values will be wide, so there is a risk of missing important pathological conditions.

Concerning iron or vitamin deficiency anemia, it is important to determine whether or not a deficiency of a particular nutrient is of such magnitude that can cause anemia. A distinction should therefore be made between a deficiency identified based on the ranges of reference values established for a definite population and a functional nutritional deficiency leading to a specific pathology, namely anemia. We propose that the determination of the cut-off point for the diagnosis of iron or vitamin deficiency anemia should be based on the analysis of the receiver-operating characteristic (ROC) curve. ROC curves are a way to analyze the accuracy of diagnostic tests and to determine the best cut-off value for distinguishing between positive and negative test results [16]. The accuracy of the test depends on how well the test separates the group being tested into those with and without the disease and is measured by the area under the ROC curve. By analyzing the ROC curve, it is possible to determine a cut-off point that would minimize the number of false negatives and false positives for a specific test. We believe that the use of this method enables the establishment of a threshold for the diagnosis of functional nutritional deficiency responsible for the development of anemia.

Therefore, the present study aimed to determine the occurrence and severity of definite etiologic types of anemia in the Polish older adults population, as well as their interdependence with morphometric characteristics of red blood cells.

## 2. Materials and Methods

### 2.1. Participants and Study Protocol

PolSenior study was a nationwide multi-disciplinary research project, conducted in 2007–2012, established for assessment of medical, psychological, social, and economic aspects of aging in Poland [17]. The study aimed to define the current status of older subjects taking into account the above dimensions of aging as well as the medical and social needs of Polish seniors.

The study was carried out on a representative group of people over 64 years of age, the comparative group consisted of people aged 55–59. The rules for the selection and composition of the study participants have been published elsewhere [17]. Among 5695 study participants, blood was collected from 4737 and urine samples from 4526 persons. Hematologic parameters measurement failed in 112 people due to technical problems (blood clots, hemolysis). In some participants of the study who underwent hematological tests, it was not possible to obtain a full set of biochemical results, mainly for technical reasons (hemolysis before centrifugation of blood collected for a clot). In relation to individual studies, the number of missing data did not exceed 10% (1.9% for the estimated glomerular filtration rate [eGFR] [*n* = 75], 6.4% for urinary albumin-to-creatinine ratio [ACR] [*n* = 255], 1.9% for serum iron [*n* = 77], 3.4% for serum ferritin [*n* = 137], 6.8% for serum folate [*n* = 271], and 7.1% for serum vitamin B_12_ [*n* = 286]), and they were not more common in the anemic population compared to non-anemic subjects.

After removing from the analysis all participants for whom a complete set of laboratory tests was not obtained, results from 4074 persons were involved in the investigation; this number consisted of 3452 older participants with age above 64 years (1632 women and 1820 men) and 622 persons from the reference group aged 55–59 years (333 women and 289 men). Participants aged above 64 were divided into six age cohorts: 65–69, 70–74, 75–79, 80–84, 85–89, and 90 or more years of age.

After collection, blood samples were delivered within 2 h to local laboratories, where complete blood count was determined with automated analyzers, and serum or plasma samples were separated and frozen. All samples were then delivered to the Central Laboratory of the Infant Jesus Clinical Hospital in Warsaw, Poland where plasma iron, plasma, and urine creatinine, and urine albumin concentrations were measured and to the Laboratory of the Department of Nephrology, Endocrinology and Metabolic Diseases at the Medical University of Silesia in Katowice, Poland for measurements of plasma levels of ferritin, vitamin B_12_ and folate.

Plasma iron concentrations were measured by the colorimetric method (Modular PPE analyzer, Roche Diagnostics GmbH, Mannheim, Germany, the limit of quantification—LoQ 5 µg/dL), ferritin with electrochemiluminescence (ECLIA) method (Cobas e411 analyzer, Roche, LoQ 0.5 ng/mL), vitamin B_12_ and folate levels with radioimmunoassay (RIA) method (SimulTRAC-SNB, MP Biomedicals, Orangeburg, NY, USA, LoQ 75 pg/mL for vitamin B_12_, and 0.6 ng/mL for folate). Plasma and urine creatinine levels were measured with the Jaffe method (Modular PPE analyzer, Roche, LoQ 0.25 mg/dL for plasma and 4.0 mg/dL for urine samples), urine albumin with the dry stripes method (Combur-Test, Roche). In cases where that method failed to detect albuminuria (albumin level below 30 mg/dL), high sensitivity immunoturbidimetric method was used (Cobas e411 analyzer, Roche, LoQ 3 mg/dL). Based on plasma creatinine level, the eGFR was calculated with the Modification of Diet in Renal Disease Study (MDRD) equation [18]. The degree of chronic kidney disease (CKD) was stratified based on the range of eGFR and ACR [19].

### 2.2. Definition, Causes, and Severity of Anemia

Anemia was defined according to the WHO criteria as blood hemoglobin (Hb) concentration < 12.0 g/dL in females and <13.0 g/dL in males [20]. Mean cell volume (MCV) of <80 fL was considered as microcytosis, while MCV > 100 fL as macrocytosis. MCV values ranging from 80 to 100 fL were classified as normocytic. The red cell distribution width coefficient of variation (RDW-CV) of more than 14.5% was considered as anisocytosis.

Cut-off points for nutritional deficiencies were determined based on three methods: (A) WHO criteria, (B) lower 2.5 percentile of the studied population, (C) functional deficiencies of iron, vitamin B_12_, and folate determined based on receiver operating characteristic (ROC) curve analysis. The exact values of cut-offs are presented in the results section and Table 3.

Subjects who had no evidence of iron, vitamin B_12_, or folate deficiency were evaluated for other causes of anemia. Subjects were considered as anemic due to chronic kidney disease if eGFR was lower than 45 mL/min/1.73 m^2^ or ACR was higher than 300 mg/g [19]. Anemia of inflammation (AI) was defined as a low serum iron level without evidence of iron deficiency (ferritin level above cut-off point) [21].

The anemia for both women and men was defined as severe when blood Hb concentration was below 10 g/dL, and as moderate when Hb was between 10 and 11 g/dL; otherwise anemia was considered as mild. Differences in the severity of anemia and MCV between the different etiological types have been determined using the ROC curve analysis only (method C).

### 2.3. Statistical Analysis

Statistical analysis was performed with Statistica data analysis software system, version 13 (TIBCO Software Inc., Palo Alto, CA, USA, 2017) upgraded with Medical Set version 4.0.67 (StatSoft, Krakow, Poland, 2020).

The frequency of anemia in the analyzed groups was compared with the use of the χ^2^ test and extended Mantel–Haenszel χ^2^ test for trend. For laboratory results, the distribution of all variables was tested for normality using the Kolmogorov–Smirnov test. Then mean values, standard deviations, and lower 2.5 percentile and upper 97.5 percentile thresholds were calculated. Comparison between two unpaired groups was made with the independent Student’s t-test and the Kruskal–Wallis test for more than two groups. In the case of significant differences between studied variables detected by the Kruskal–Wallis test, a post hoc Duncan test was performed.

To establish the relationship between anemia and individual nutrients, the correlation coefficient between the level of hemoglobin and the level of folate, vitamin B_12_, iron, and ferritin was calculated.

The optimal cut-off levels that differentiated patients with and without nutritional deficiency anemias at the highest sensitivity and specificity rates were determined using Youden’s index calculated by sensitivity + specificity − 1. The accuracy of the test was assessed based on the area under the ROC curve (AUC). The AUC values > 0.9, 0.7 to 0.9, and 0.5 to 0.7 corresponded to the high, moderate, and low diagnostic accuracy of the test, respectively [22].

The frequency of specific nutrient deficiency (classified according to three above-described methods) among anemic and non-anemic participants was compared with the use of the χ^2^ test.

## 3. Results

### 3.1. Characteristics of the Study Group and Morphometric Classification of Anemia

The mean age of 3452 participants of the study was 78.6 ± 8.6 years (range: 65–104 years). The detailed characteristics of the studied group are presented in Table 1. A significantly higher incidence of anemia was observed in men than in women and the frequency of anemia increased with age, reaching 44.8% in nonagenarian men. Anemia was also more common in people with CKD and dementia but less frequent in the obese. In women, anemia was more common in individuals with Parkinson’s disease, chronic pain, and post-stroke. Participants of both genders with anemia were characterized by lower serum levels of iron, ferritin, and higher serum concentration of folic acid.

According to the MCV values, normocytosis was the dominant form of anemia in all age cohorts, occurring in 88.4% of the entire studied population with anemia, while microcytosis was observed in 6.6%, and macrocytosis in 5.0% of anemic persons. In women, the percentage of normocytosis in all anemia cases was significantly lower than in men (84.7% vs. 89.3%; *p* < 0.05) while microcytosis was significantly higher (10.0% vs. 5.3%; *p* < 0.05).

**Figure 1:**
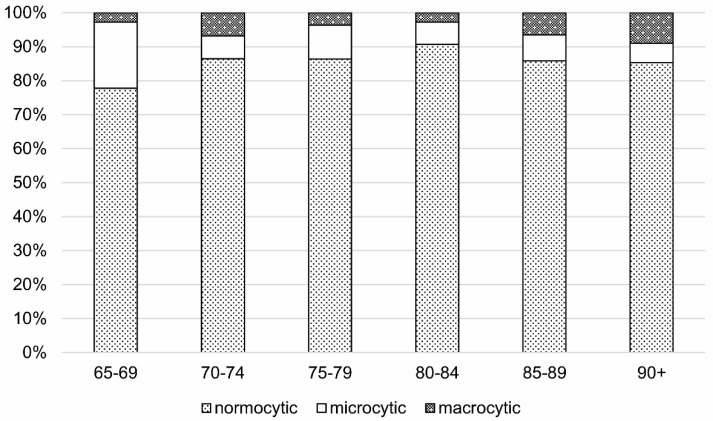
The percentage rate of normocytic, microcytic, and macrocytic anemia in age cohorts in the PolSenior study.

We did not observe significant differences between age cohorts concerning the percentage of normo-, micro-, and macrocytic anemias, with two exceptions: the youngest cohort (65–69 years old) was characterized by the higher percentage of microcytic anemia (*p* < 0.01), and the oldest cohort (90 years or older) had a higher percentage of macrocytic anemia compared to other age groups (*p* < 0.05) (Figure 1).

Anisocytosis was observed in 32.1% of normocytic anemias, in 82.9% of microcytic anemias, and 45.8% of macrocytic anemias (*p* < 0.0001). There were no significant differences in the prevalence of anisocytosis among anemic participants between both sexes (women—39.3%; men—34.7%), as well as between age cohorts (with percentage values in successive cohorts: 16.7%; 41.7%; 44.6%; 27.8%; 35.8%; 38.8%).

### 3.2. Etiology of Anemia in the Elderly Population

In the anemic older participants, we have observed a positive correlation between blood hemoglobin and plasma ferritin concentrations (r = 0.154; *p* < 0.05), as well as between Hb and plasma iron levels (r = 0.394; *p* < 0.05). There were no significant correlations between Hb and plasma vitamin B_12_, as well as between Hb and plasma folate concentrations.

The lower 2.5 percentiles thresholds for plasma levels of nutritional compounds in the studied population were as follows: 1.6 ng/mL for folate, 75 pg/mL for vitamin B_12_ (at the level of quantification), 25 ng/mL for ferritin, and 35.1 µg/dL for iron.

**Figure 2:**
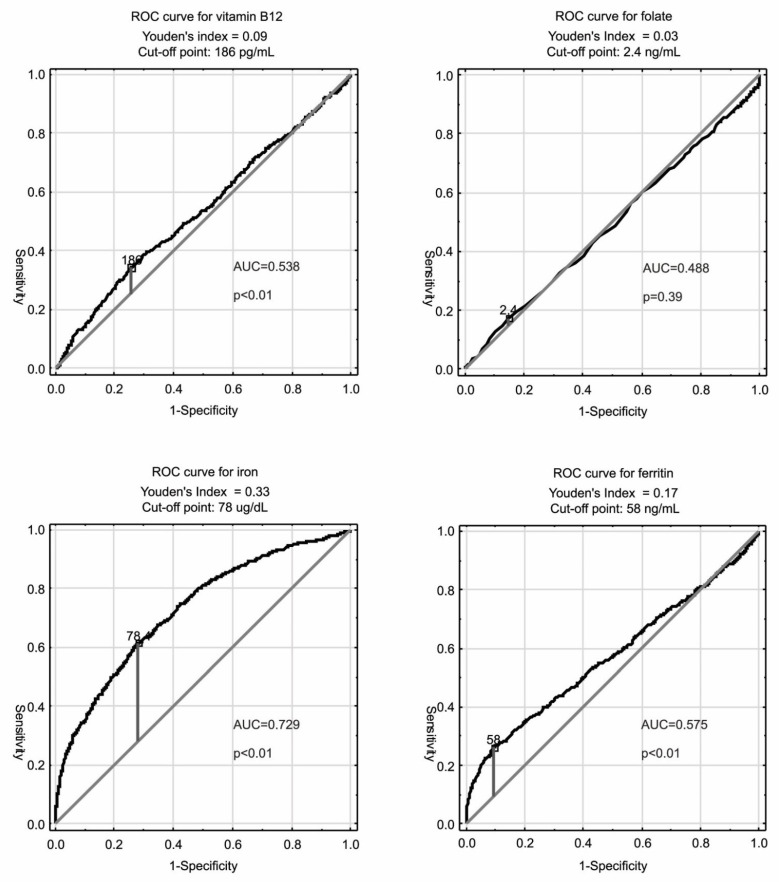
ROC curves for interdependency between anemia and plasma levels of vitamin B_12_, folate, iron, and ferritin in the PolSenior study. ROC—receiver operating characteristic; AUC—area under ROC curve.

ROC curve analysis was performed for relationships of anemia with plasma levels of vitamin B_12_, folate, iron, and ferritin. Based on this analysis, the cut-off points were established as follows: for ferritin 58 ng/mL; for iron 78 µg/dL; for folate 2.4 ng/mL; for vitamin B_12_ 186 pg/mL. ROC curves and AUC are presented in Figure 2. Etiologic classification of different types of anemia with cut-off points established according to three different methods, namely the WHO criteria, lower 2.5 percentile of studied population, and ROC curve analysis are presented in Table 2.

When using the WHO criteria, a comparison of the percentage of nutritional deficiencies in participants with and without anemia (not shown in Table 2) shows the equal incidence of folate deficiency (28.8% vs. 29.7%, not significant), a higher incidence of vitamin B_12_ deficiency (38.3% vs. 29.6%, *p* < 0.0001) and a remarkably higher incidence of iron deficiency (3.3% vs. 0.1%, *p* < 0.0001) in persons with anemia. In the case of using the lower 2.5 percentile of the studied population limit, no significant difference was found in the frequency of folate deficiency in persons with and without anemia (2.2% vs. 2.0%; not significant). No significant differences in these two groups were also observed concerning vitamin B_12_ deficiency (3.3% vs. 2.3%, respectively), but the difference was remarkable for iron deficiency (9.3% vs. 1.1%, respectively, *p* < 0.0001). In the classification of the causes of anemia based on the ROC curve analysis, folic acid deficiency occurred at the same frequency in anemic and non-anemic subjects (14.4% vs. 12.8%, not significant); however, vitamin B_12_ deficiency (33.6% vs. 25.1%, *p* < 0.0001) and iron deficiency (24.9% vs. 9.1%, *p* < 0.0001) were much more common in persons with anemia.

Differences between normo-, micro-, and macrocytosis in definite types of anemia are presented in Table 3. Among the participants with anemia caused by iron and vitamin deficiency, normocytosis was less common (*p* < 0.01) than in persons with non-deficiency anemias. In anemia caused by iron deficiency (IDA), occurring either alone or in combination with folate or vitamin B_12_ deficiency, microcytosis was more common, and normocytosis was less common than in other types of anemia (*p* < 0.0001). In contrast, macrocytosis was more common in vitamin B_12_ deficiency compared to other types of anemia (*p* < 0.01). Such tendency was not observed in anemia caused by folate deficiency, both occurring alone or in combination with vitamin B_12_ deficiency. Anemia caused by inflammation (AI) was characterized by the highest rate of normocytosis compared to other types of anemia (*p* < 0.01). Anisocytosis was more common in deficiency than non-deficiency anemia cases (44.8% vs. 25.8%, respectively, *p* < 0.0001). Among the normocytic anemias, anisocytosis was more common in subjects with nutritional deficiencies than in non-deficient anemia cases (39.1% vs. 24.3%, respectively, *p* < 0.001).

In 40% of anemic subjects classified as related to nutrient deficiency, we found the coexistence of non-deficiency cause (Table 4). In 62.9% of such coexistence, the deficiency was accompanied by CKD, in 24.2% by AI, and in 12.9% by both these non-deficiency conditions. Among the different causes of deficiency, the coexistence of the non-deficiency factors was more common in folate, vitamin B_12_ deficiency anemia, and less frequent in complex deficiency of iron and folate or vitamin B_12_. Among the iron-deficiency anemia subjects, we did not observe a significant difference in the frequency of coexistence of non-deficiency factors between normocytic (32.8%, *n* = 19) and microcytic cases (31.3%, *n* = 5). We observed an increasing percentage of coexistence of non-deficiency cause among deficiency anemic subjects in consecutive age cohorts, from 10.4% in the youngest to 33.1% in the oldest cohort (χ^2^ = 20.4; *p* < 0.0001).

### 3.3. The Severity of Anemia in the Elderly Population

**Figure 3:**
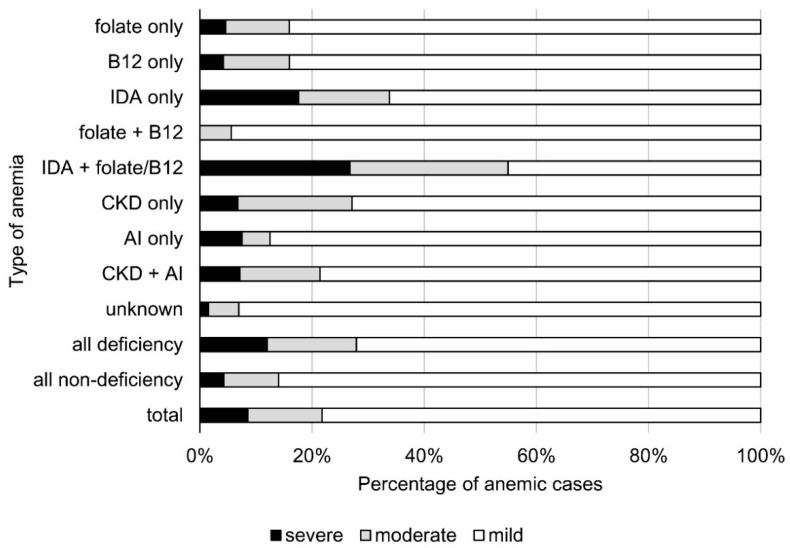
The proportion between severe (hemoglobin—Hb < 10 g/dL), moderate (Hb: 10–11 g/dL), and mild anemia in defined types of anemia (classified according to receiver operating characteristic curve analysis) in the PolSenior study. AI—anemia of inflammation; CKD—chronic kidney disease; IDA—iron deficiency anemia.

Characteristics of mild, moderate, and severe anemia concerning gender, age cohorts, MCV, and etiology are presented in Table 5 and the proportion between mild, moderate, and severe anemia in different etiological types are shown in Figure 3.

The deficiency anemia was characterized by the overall lower rate of mild cases than the non-deficiency anemia (72% vs. 86%; *p* < 0.0001). The lowest rate of mild anemia was related to iron deficiency, both in situations where the deficiency was present alone or in combination with vitamin B_12_ and folate deficiencies (*p* < 0.01). Compared to IDA, the prevalence of mild anemia caused by folate and vitamin B_12_ deficiency anemia was higher, both when these two latter occurred alone and in combination. Among the non-deficiency anemias, the highest prevalence of mild cases was attributed to anemia of unknown causes (*p* < 0.0001).

In addition to the data presented in Table 5 and Figure 3, we have observed a lower proportion of mild anemia (Hb < 11 g/dL) among anemic women compared to anemic men (67.9% vs. 84.0%; *p* < 0.0001). The percentage of mild cases among all cases of anemia in the subsequent age cohorts was as follows: 65-69 years old 77%, 70–74 years old 87%, 75–79 years old 74%, 80–84 years old 86%, 85–89 years old 76%, 90 and older 74%. There were no significant differences between age cohorts except for the 80–84 years cohort, where there were proportionally more cases of mild anemia (*p* < 0.05). There were significantly more subjects with microcytic and fewer with normocytic anemia in the group of persons with moderate-to-severe anemia compared to those with mild anemia (both *p* < 0.00001, Table 4).

## 4. Discussion

### 4.1. Prevalence of Anemia

To the best of our knowledge, the only population-based survey on a nationally representative group of older people that includes the etiological classification of anemia is the NHANES III study performed in the USA in 1988–1994 and published in 2004 [23]. In this population, anemia was found in 11% of men and 10% of women aged 65 years or older. According to the classification criteria adopted by the NHANES III research group, one-third of anemia cases were due to nutritional deficiencies, one-third were due to chronic kidney disease and chronic inflammation, and one-third had an unknown etiology. Among deficiency anemia, most cases were associated with iron deficiency [23]. Other nationwide population studies including geriatric cohorts did not consider the etiological classification in the analysis of the prevalence of anemia or were focused only on one of the anemia causes, such as iron deficiency [24,25,26]. Some population studies, limited to the local group of older adults, have undertaken an analysis of the frequency of particular types of anemia. An example is the “Health and Anemia” research conducted on a large group of nearly nine thousand older people living in Biella in Italy, where among over 1200 anemic individuals (14.2% of the study group) the deficiency anemia constituted 26%, CKD-related anemia 15%, and AI 17% of all anemia cases. Noteworthy, in that study, about 15% of anemic cases were associated with thalassemia, which is common in the Mediterranean countries. For the remaining 26% of anemia, the etiology was not found [27].

In our previous publication, the standardized prevalence of anemia in the Polish older population was estimated at 10.8%, which is comparable to values obtained in NHANES III [23]. Similarly, in the InCHIANTI Study performed on older inhabitants of the Chianti region in Italy, the prevalence of anemia was 11.3% [4]. We demonstrated that in concordance with the above-mentioned studies, the frequency of anemia increased with age also in the Polish elderly population and reached 37.7% in the oldest cohort aged 90 years and older [28]. The tendency to the increasing prevalence of anemia in subsequent age cohorts of the elderly population is confirmed by another study performed on the population of Polish centenarians, where the prevalence of anemia reached 29% in women and 57% in men [29]. In our study significant increase in the frequency of anemia has been observed especially from the ninth decade of life. One of the possible explanations is the coexistence of multiple etiological factors of anemia, as reflected in our data showing that the prevalence of multifactorial anemia increases in subsequent age cohorts. The significantly higher prevalence of anemia in the ninth and tenth decades of life may also be because, after the age of eighty, the bone marrow is characterized by decreased cellularity, which may be partly attributed to an increase in apoptosis [30].

The observed higher incidence of anemia in men is most likely due to the adoption of different cut-off points for blood Hb concentration according to the WHO criteria. With age, there is a decrease in the gender differences in certain physiological processes that affect the concentration of blood hemoglobin. The cessation of menstrual blood loss in postmenopausal women and the reduced advantage of erythropoiesis-stimulating factors (e.g., declining androgen production) in men are among the major causes [31]. That’s why some researchers suggested the need for applying the same reference value of blood hemoglobin concentration, 12 g/dL, in older men as in women [32]. On the other hand, it is known that mortality in older men increases not only when the blood hemoglobin level is lower than 12 g/dL, but also in subjects with blood hemoglobin levels in the range between 12 and 13 g/dL [33,34]. Although this topic remains disputable, the latter data seem to support the WHO criteria for the diagnosis of anemia in the elderly population, Hb < 12 g/dL for women and Hb < 13 g/dL for men [20].

### 4.2. Criteria of Etiologic Classification of Anemia

The cut-off points for the diagnosis of iron, vitamin B_12_, and folate deficiencies established by WHO in the 1960s were based on the younger population and do not take into account the age-specific physiological changes related to the absorption, utilization, and metabolism of nutrients involved in erythropoiesis [20]. Therefore, it is questionable whether we should apply these criteria to the elderly population for the diagnosis of nutritional deficiencies. The application of the WHO criterion for the diagnosis of iron deficiency (serum ferritin concentration < 15 ng/mL) in relation to our study group resulted in a significant underestimation of this type of anemia compared to other studies. IDA alone or in combination with folate or vitamin B_12_ deficiency was represented in our study by 3.3% of all cases with anemia, while in NHANES III by 20%, and in the Italian “Health and Anemia” study by 16% of anemic individuals [23,27]. On the other hand, the application of the WHO criteria for folate (serum folate concentration < 3 ng/mL) and vitamin B_12_ (serum vitamin B_12_ concentration < 200 pg/mL) deficiency suggests an overestimation of the related anemia in our study group. According to WHO criteria, these two deficiencies were associated with more than half of all anemia cases in our participants, while in the NHANES III study it was about 15%, and in the “Health and Anemia” only 10% [23,27]. It is worth noting, however, that the researchers of NHANES III and “Health and Anemia” studies used different parameters for the classification of nutritional deficiencies and anemia of inflammation [23,27]. The exact values of those criteria were presented in Table 6 and compared to criteria used in the “PolSenior” study.

In turn, the application of the lower 2.5 percentile criterion of the studied population (serum ferritin < 25 ng/mL for IDA, serum folate < 1.6 ng/mL for folate deficiency, serum B_12_ < 75 pg/mL for vitamin B_12_ deficiency) leads to an underestimation of all deficiency anemias, especially those resulting from folate and vitamin B_12_ deficiency (2.2% and 5.0%, respectively, of all anemia cases) with a very high share of unexplained anemia (51.6%). Since the criteria of nutritional deficiencies based on the young population do not apply to old people, and the criteria originating from the distribution of nutritional parameters in the geriatric population are burdened with an underestimation error, we need to create cut-off points for anemia-specific functional nutritional deficiencies. For this purpose, we used a method based on the ROC curve analysis. It allowed the establishment of cut-off points for the diagnosis of functional deficiency of folate (serum folate < 2.4 ng/mL), vitamin B_12_ (serum B_12_ < 186 pg/mL), and iron (serum ferritin < 58 ng/mL). Based on these criteria, we determined the percentage of each type of anemia (Table 2).

Some reservations to the use of this method to determine the etiological classification of anemia may be raised by the fact that the analysis of the area under the curve showed moderate accuracy (0.73) only for serum iron levels, while for levels of vitamin B_12_ and ferritin accuracy was low (0.54 and 0.57 respectively), and for serum folate concentration it was statistically insignificant and lower than 0.5 (Figure 2). However, it should be kept in mind that the analysis of ROC curves is primarily used in the evaluation of tests designed to detect a specific disease to find the optimal marker and establish a cut-off point differentiating between people in whom a disease may occur with a high probability from those who are most likely free from this pathology. In our opinion, the application of the ROC curve analysis to the adopted goal of the etiological classification of anemia does not have to meet the criteria of high accuracy.

A striking observation in our research is that there is no association between serum folate levels and anemia, which is concordant with the observed very low area under the ROC curve for folate. Using different cut-off points to diagnose folate deficiency, we found no significantly higher rates of anemia in participants with deficiency compared to those without. Moreover, the mean serum folate level in anemic persons turned out to be higher compared to non-anemic. It may, therefore, be questioned whether folate deficiency is still a significant etiological factor in anemia in older adults. These doubts appeared also in the previously cited studies, where, in old age, there was no effect of folate deficiency on hematological parameters or functional status [9].

In our studies, we adopted the following criteria for the classification of anemia as related to kidney function: eGFR < 45 mL/min/1.73 m^2^ or ACR > 300 mg/g. Although current criteria for the diagnosis of CKD in adults include persistent signs of kidney damage, or a GFR below 60 mL/min/1.73 m^2^ or elevated urinary albumin-to-creatinine ratio above 300, the latest reports indicate that unlike in young adults where mortality is associated with impaired renal function increases already at GFR < 75 mL/min/1.73 m^2^, in older adults it increases only at GFR < 45 mL/min/1.73 m^2^ [19]. This is probably the reason for the slightly lower incidence of anemia related to CKD observed in our study compared to the “Health and Anemia” study, where CKD was diagnosed based on current criteria [27]. On the other hand, the frequency of CKD-related anemia in the NHANES III was lower than in our study, but they adopted the threshold of GFR < 30 mL/min/1.73 m^2^ as a diagnostic criterion for impaired renal function [23].

### 4.3. MCV and Etiologic Classification of Anemia

The vast majority of anemia cases in our study had MCV values within the reference range. This applied not only to non-deficiency anemia but also to that associated with iron, vitamin B_12_, and folate deficiencies. Moreover, while microcytosis was more common in people with IDA compared to other types of anemia, and macrocytosis was more common in patients with vitamin B_12_ deficiency anemia, macrocytosis was not found to be more frequent in folate-deficient patients than in other types of anemia. This observation is consistent with a study conducted on a group of over 46,000 residents of Calgary, Canada, and undermines the usefulness of MCV in the differential diagnosis of the causes of anemia in older patients, especially concerning folate-deficient anemia [35].

However, it should be mentioned that among the participants of the PolSenior study with normocytic deficiency anemia nearly 40% had anisocytosis. This fact was mostly related to anemia with coexisting iron and vitamin B_12_/folate deficiencies (57%) and anemia with isolated iron deficiency (49%). It can therefore be assumed that in many cases of anemia in old age, nutritional deficiency affects the newly formed red blood cells, changing their volume (which is reflected in anisocytosis), but it is not advanced enough to cause a shift of MCV beyond the reference values towards microcytosis in iron deficiency or macrocytosis in vitamin B_12_/folate deficiency. Hence, the analysis of the causes of anemia must not be based solely on MCV, but on other red cell parameters, especially on the RDW.

Similar to the NHANES III and “Health and Aging” study, the subjects of the PolSenior study were assigned either to the group of deficient or non-deficient anemia [23,27]. Such a division means that cases of anemia of complex etiology, where nutritional deficiencies coexist with non-deficient causes—CKD or AI are not taken into account. Such multifactorial anemia, typical for old persons, may cause the ambiguous morphological characteristics of erythrocytes and significantly reduce the diagnostic usefulness of such simple parameters as MCV. As can be seen from the data presented in Table 5, the coexistence of the non-deficiency factor with a deficiency cause of anemia is very common and significantly more often applies to normocytic anemia compared to micro- and macrocytic anemia. This suggests that in some anemic subjects classified as deficient, the coexisting non-deficiency factor (e.g., renal function impairment or inflammation) may play a greater role in the etiopathogenesis of anemia than the deficiency itself. In the study performed in the city of Dordrecht area, the Netherlands, performed on the group of over 4000 anemic persons aged >50, the anemia related to multiple causes was found in 22% of subjects and the coexistence of vitamin B12 or folate deficiency with other etiologies was the most common [36]. These results are consistent with our observation, where the coexistence of non-deficient etiology was more often observed in vitamin B12 or folate deficiency compared to iron deficiency.

The youngest cohort of old subjects in our study had a higher percentage of microcytic anemia, and the oldest cohort was characterized by a higher percentage of macrocytic anemia. One of the possible causes may be related to the preservation of the possibility of proliferative bone marrow reaction to iron deficiency in the youngest group of older people and the exhaustion of this mechanism in nonagenarians [37]. On the other hand, experienced hematologists point out that the bone marrow of 90-year-olds is often cell-rich, both in microcytic iron deficiency and in macrocytic anemias related to vitamin B_12_ deficiency.

### 4.4. Anemia of Unknown Etiology

NHANES III study group reported nearly one-third of all anemia cases as unexplained, “Health and Anemia” study failed to explain 26% of anemic cases [23,27]. In the analysis of the causes of anemia in patients hospitalized in the geriatric unit, 17% of all cases were of unknown etiology [38]. In turn, in the research of the National Geriatrics Research Consortium conducted on residents of nursing homes, anemia due to unexplained reasons constituted as much as 43% of all cases [39].

In our study, nearly 130 anemia cases remained unexplained, which represents 22% of anemic participants. In a closer analysis of those cases, we found four cancer survivors and four persons suffering from active malignant neoplasm, eight persons had hypothyroidism, two had thyrotoxicosis, and two were infected by hepatitis C virus. It should be noted, however, that the frequency of these disorders in the group with unexplained anemia did not differ from the frequency in the entire study population. In a further four individuals with unexplained anemia, the presence of myelodysplastic syndrome (MDS) may be presumed as a possible cause of anemia because, apart from anemia, they present with leucopenia or thrombocytopenia or both, although it is impossible to verify this assumption without conducting bone marrow assessment including cytogenetic studies [40]. The frequency of MDS reported here is certainly underestimated. The older the cohort, the more the number of MDS increases, which initially manifests most often with anemia without leucopenia or thrombocytopenia, which usually join later [41]. Such underestimation also takes place in clinical practice, which may result from non-specific symptoms and the need for complex cytological and cytogenetic diagnostics.

The remaining 106 cases of unexplained anemia (81%) should probably be attributed to the aging process, in particular age-associated decline in endocrine function of the kidneys resulting in a reduced erythropoietin secretion and, possibly, age-associated reduction in androgen levels in both males and females, accounting for a decline in hemoglobin level [42].

### 4.5. Severity of Anemia

For most of the study participants with anemia, the disease was mild and Hb level was 11 g/dL or higher. Despite the higher prevalence of anemia among older cohorts, the severe anemia rate in older groups was comparable to younger cohorts. The rate of moderate-to-severe anemia was higher among women compared to men. The likely cause of this observation is that a large group of anemic men had hemoglobin levels between 11 and 13 g/dL, making their anemia classified as mild, while for women it is enough for the hemoglobin to drop by 1 g/dL to cause anemia classified as moderate.

Iron deficiency was the major cause of moderate-to-severe anemia cases, especially when it was combined with other nutritional deficiencies (Figure 3). In contrast, anemia caused by a folate and vitamin B_12_ deficiency was usually mild. Among the non-deficient causes, the highest rate of mild anemia was represented by unexplained anemia. This is in line with the observations of other investigators, where cases of unexplained anemia were usually associated with reductions in blood Hb levels of less than 1 g/dL [23,27].

### 4.6. Limitations

Our study has some limitations. First, in the classification of anemia caused by iron deficiency, the diagnosis was based on ferritin levels. In the HNANES III and “Health and Aging” studies, apart from the assessment of serum iron and ferritin levels, transferrin saturation was also taken into account, and in the first of the two studies, the concentration of zinc protoporphyrin in erythrocytes was also measured [23,27]. Our study did not assess the concentration of transferrin, and hence its saturation. As concluded by the Joint WHO/Centers for Disease Control and Prevention (CDC) Technical Consultation on the Assessment of Iron Status at the Population Level in 2004, transferrin saturation is characterized by lower sensitivity/specificity than serum ferritin in the prediction of iron deficiency [13]. According to WHO recommendations on assessing the iron status of populations, measurements of serum ferritin and soluble transferrin receptor (sTfR) provide the best approach to measuring the iron status [13]. We did not examine the concentration of sTfR, which may help identify an iron deficiency in patients who have elevated levels of ferritin due to inflammation. However, some studies suggest that the measurement of sTfR does not provide sufficient additional information to ferritin to warrant routine use when diagnosing IDA [43].

The second limitation is related to the criteria for diagnosing folate deficiency. We measured the concentration of folate in the serum. A more accurate method is to measure the concentration of folate in the red blood cells, and this was the method used in the NHANES III study [23]. However, it should be remembered that the participants of our study were tested in their own homes and blood was collected there. Local laboratories only performed complete blood count, and the central laboratory measured the biochemical parameters. Therefore, the determination of the RBC components would be a very serious organizational obstacle and could be associated with a much lower implementation rate of the entire PolSenior research program.

Concerning folate and vitamin B_12_ deficiency, some researchers postulate that serum homocysteine levels are measured to detect latent deficiency of these two nutrients. On the other hand, there are reports that in patients with chronic obstructive pulmonary disease (COPD) who are a population at risk of folate and vitamin B_12_ deficiency, reliability of the homocysteine concentration as a biomarker of folate or vitamin B_12_ depletion is not satisfactory, so their deficiency cannot be predicted by the occurrence of high serum homocysteine [44].

Finally, the methodology of classification of IDA and AI adopted by us (based on iron and ferritin concentrations) implies the inability of the combination of these two types of anemia. In fact, these two etiological factors can coexist in many people with anemia, although the condition can be difficult to diagnose because ferritin is on one hand an iron-binding protein and on the other hand an acute-phase protein. The serum hepcidin concentration would be the best indicator to differentiate IDA from AI, but the gold standard for measurement of this protein in clinical practice is still lacking [45].

## 5. Conclusions

- In the etiological classification of anemia, it is necessary to adopt the age-specific criteria for the diagnosis of functional deficiency of nutrients such as iron, folates, and vitamin B_12_, because not every deficiency of these nutrients in older subjects may cause anemia.
- About half of the cases of anemia in old age are caused by nutritional deficiencies. Of the remaining causes, chronic kidney disease and chronic inflammation play a significant role. Every fifth case of anemia in old age has no clear explanation.
- MCV analyzed alone is of limited importance in the differential diagnosis of types of anemia in older adults, since most of the deficient anemia in the geriatric population remains normocytic. Therefore, it should be analyzed together with other red cell parameters, especially with RDW, which may increase the diagnostic possibilities of the morphological examination.
- In the older population with anemia, many subjects have a complex disease etiology resulting from the coexistence of deficiency and non-deficiency causes. Extensive laboratory analysis in old anemic patients may reveal many various combinations of etiologies of anemia and help to optimize the therapy.
- Iron deficiency anemia is more severe than folate and vitamin B_12_ deficiency anemia. Severe anemia resulting from unexplained etiology is rare.
- Folic acid deficiency has little significance in the pathogenesis of anemia in old age.

## Figures and Tables

**Table 1:** Characteristics of female and male participants of the PolSenior study with or without anemia. Quantitative data presented as mean values ± standard deviation.

Cohorts	Females		Males	
	Anemic	Non-Anemic	Anemic	Non-Anemic
Age [years]	83.0 ± 8.8 ^a^	77.6 ± 8.4	84.2 ± 7.8 ^a^	77.6 ± 8.1
65–69 years [% (*n*)]	5.9 (18)	94.1 (289)	4.4 (13)	95.6 (281)
70–74 years [% (*n*)]	7.8 (26)	92.2 (309)	11.8 (41) ^b^	88.2 (306)
75–79 years [% (*n*)]	9.9 (28)	90.1 (254)	14.0 (45) ^b^	86.0 (276)
80–84 years [% (*n*)]	12.3 (30)	87.7 (213)	21.6 (63) ^b^	78.4 (228)
85–89 years [% (*n*)]	17.4 (42)	82.6 (200)	32.0 (105) ^b^	68.0 (223)
90 and over [% (*n*)]	29.1 (65)	70.9 (158)	44.8 (107) ^b^	55.2 (132)
Total [% (*n*)]	12.8 (209)	87.2 (1423)	20.5 (374) ^b^	79.5 (1446)
**Comorbidity**				
Gastrointestinal ulcer [% (*n*)]	8.6 (16)	8.5 (112)	12.4 (41)	10.8 (148) ^b^
Parkinson’s disease [% (*n*)]	2.7 (5) ^a^	1.6 (21)	2.4 (8)	3.1 (42)
Past stroke [% (*n*)]	12.3 (23) ^a^	5.3 (70)	9.4 (31)	7.5 (103)
Dementia [% (*n*)]	48.3 (101) ^a^	31.5 (448)	37.4 (140) ^a^^b^	23.3 (337)
Obesity [% (*n*)]	28.2 (59) ^a^	38.9 (554)	15.2 (57) ^ab^	26.8 (388) ^b^
Visceral obesity [% (*n*)]	75.6 (158) ^a^	84.3 (1200)	58.0 (217) ^ab^	72.4 (1047) ^b^
Underweight [% (*n*)]	1.0 (2)	0.7 (10)	1.6 (6)	1.4 (20)
History of cancer [% (*n*)]	4.8 (10)	5.1 (72)	6.5 (24)	4.3 (62)
Chronic pain [% (*n*)]	58.3 (109) ^a^	47.2 (626)	39.7 (131) ^b^	36.3 (498) ^b^
Chronic hepatitis [% (*n*)]	4.8 (10)	2.7 (38)	3.2 (12)	3.2 (46)
Chronic kidney disease [% (*n*)]	33.0 (69) ^a^	14.1 (200)	31.6 (118) ^a^	8.4 (122) ^b^
Thyrotoxicosis [% (*n*)]	4.8 (10)	3.2 (45)	2.7 (10)	1.7 (24) ^b^
Hypothyroidism [% (*n*)]	7.7 (16)	9.3 (133)	5.6 (21)	5.0 (72) ^b^
**Parameters**				
White blood cells [10^3^/µL]	6.08 ± 1.68 ^a^	6.39 ± 2.07	6.6 ± 3.2 ^b^	6.7 ± 1.9 ^b^
Red blood cells [10^6^/µL]	3.83 ± 0.41 ^a^	4.54 ± 0.40	4.0 ± 0.4 ^ab^	4.8 ± 0.4 ^b^
Hemoglobin [g/dL]	11.0 ± 1.2 ^a^	13.6 ± 1.0	11.8 ± 1.1 ^ab^	14.6 ± 1.0 ^b^
Hematocrit [%]	33.5 ± 3.5 ^a^	40.7 ± 3.2	35.7 ± 3.4 ^ab^	43.3 ± 3.4 ^b^
MCV [fL]	87.9 ± 8.6 ^a^	89.6 ± 5.6	90.5 ± 7.7 ^b^	91.0 ± 5.4 ^b^
MCH [pg]	28.8 ± 3.4 ^a^	30.0 ± 1.9	30.1 ± 3.0 ^ab^	30.8 ± 1.9 ^b^
MCHC [g/dL]	32.4 ± 3.2 ^a^	33.2 ± 2.3	32.9 ± 2.7 ^a^	33.5 ± 2.1 ^b^
Platelets [10^3^/µL]	250 ± 93 ^a^	230 ± 68	219 ± 81 ^ab^	208 ± 73 ^b^
Serum creatinine [mg/dL]	1.03 ± 0.49 ^a^	0.85 ± 0.24	1.2 ± 0.6 ^ab^	1.0 ± 0.3 ^b^
Serum iron [µg/dL]	66.8 ± 28.2 ^a^	91.2 ± 29.1	74.8 ± 32.1 ^ab^	103.7 ± 32.7 ^b^
Serum folate [ng/mL]	6.5 ± 9.0 ^a^	5.0 ± 4.2	5.3 ± 6.2 ^a^	4.4 ± 3.7 ^b^
Serum vitamin B_12_ [pg/mL]	289 ± 249	304 ± 250	288 ± 282	287 ± 183 ^b^
Serum ferritin [ng/mL]	164 ± 199 ^a^	171 ± 129	227 ± 251 ^ab^	260 ± 216 ^b^
eGFR-MDRD [mL/min/1.73 m^2^]	55.2 ± 20.5 ^a^	64.5 ± 17.0	58.3 ± 20.7 ^a^	70.2 ± 17.3 ^b^
ACR [mg/g]	136 ± 701 ^a^	33 ± 161	120 ± 461 ^a^	39 ± 209

^a^ *p* < 0.05 vs. non-anemic; ^b^ *p* < 0.05 vs. females; ACR—urine albumin to creatinine ratio; eGFR-MDRD—glomerular filtration rate estimated by Modification of Diet in Renal Disease Study equation; MCH—mean cell hemoglobin; MCHC—mean cell hemoglobin concentration; MCV—mean cell volume.

**Table 2:** Etiologic classification of anemias in the PolSenior study with cut-offs established according to different methods.

Method	WHO		Lowest 2.5 Percentile		ROC	
Type of Anemia (Parameter [Unit])	Cut-Offs	% (*n*)	Cut-Offs	% (*n*)	Cut-Offs	% (*n*)
**Anemia with Nutritional Deficiency**						
Iron only (serum ferritin [µg/L])	<15	0.9 (5)	<25	9.1 (53)	<58	12.7 (74)
Folate only (serum folate [ng/mL])	<3	18.4 (107)	<1.6	2.2 (13)	<2.4	7.5 (44)
B_12_ only (serum B_12_ [pg/mL])	<200	26.9 (157)	<75	5.0 (29)	<186	20.4 (119)
Folate and B_12_	see above	9.4 (55)	see above	0.0 (0)	see above	3.1 (18)
Iron and folate or B_12_ or both	see above	2.4 (14)	see above	0.2 (1)	see above	12.2 (71)
All deficiency anemias	see above	58.0 (338)	see above	16.5 (96)	see above	55.9 (326)
**Anemia without Nutritional Deficiency**						
CKD only (eGFR < 45 mL/min/1.73 m^2^ or ACR > 300 mg/g)	see in 1st column	8.9 (52)	see in 1st column	26.1 (152)	see in 1st column	10.1 (59)
AI only (serum iron [µg/dL]; serum ferritin [µg/L])	<60; ≥15	8.7 (51)	<35.1; ≥25	2.6 (15)	<78; ≥58	6.9 (40)
CKD/AI	see above	4.8 (28)	see above	3.3 (19)	see above	4.8 (28)
Unknown	-	19.6 (114)	-	51.6 (301)	-	22.3 (130)
All non-deficiency anemias	see above	42.0 (245)	see above	83.5 (487)	see above	44.1 (257)
**Total**	-	100 (583)	-	100 (583)	-	100 (583)

ACR—urine albumin to creatinine ratio; AI—anemia of inflammation; CKD—chronic kidney disease; eGFR—estimated glomerular filtration rate; ROC—receiver operating characteristic; WHO—World Health Organization.

**Table 3:** Normo-, micro-, and macrocytosis in definite types of anemia (classified according to receiver operating characteristic analysis) in the PolSenior study.

Type of Anemia	Normocytic % (*n*)	Microcytic % (*n*)	Macrocytic % (*n*)
**Anemia with Nutritional Deficiency**			
Folate only	7.8 (40)	4.9 (2)	6.5 (2)
B_12_ only	20.4 (104)	2.4 (1) ^a^	45.2 (14) ^a^
IDA only	11.4 (58) ^a^	39.0 (16) ^a^	0.0 (0)
Folate + B_12_	3.3 (17)	0.0 (0)	3.2 (1)
IDA + folate/B_12_	10.6 (54) ^a^	36.6 (15) ^a^	6.5 (2)
All deficiency anemias	53.4 (273) ^a^	82.9 (34) ^a^	61.3 (19)
**Anemia without Nutritional Deficiency**			
CKD only	10.8 (55)	2.4 (1)	9.7 (3)
AI	7.8 (40) ^a^	0.0 (0)	0.0 (0)
CKD + AI	4.9 (25)	2.4 (1)	6.5 (2)
Unknown	23.1 (118)	12.2 (5)	22.6 (7)
All non-deficiency anemias	46.6 (238) ^a^	17.1 (7) ^a^	38.7 (12)
**Total**	100.0 (511)	100.0 (41)	100.0 (31)

^a^ *p* < 0.05 compared to other types of anemia; AI—anemia of inflammation; CKD—chronic kidney disease; IDA—iron deficiency anemia.

**Table 4:** Deficiency anemias and coexisting non-deficiency anemia causes (classified according to receiver operating characteristic analysis) in the PolSenior study.

Deficiency Anemia	Coexisting Non-Deficiency Cause % (*n*)	Deficiency Cause(s) Only % (*n*)
Normocytic ^a^	88.6 (117)	80.4 (156)
Microcytic	6.8 (9)	12.9 (25)
Macrocytic	4.5 (6)	6.7 (13)
Folate only ^a^	63.6 (28)	36.4 (16)
Vitamin B_12_ only ^a^	48.7 (58)	51.3 (61)
IDA only	32.4 (24)	67.6 (50)
Folate + B_12_	44.4 (8)	55.6 (10)
IDA + folate/B_12_^a^	19.7 (14)	80.3 (57)
All deficiency anemias	40.5 (132)	59.5 (194)

^a^ *p* < 0.05 vs. other types of anemia; IDA—iron deficiency anemia.

**Table 5:** Characteristics of severe (hemoglobin [Hb] < 10 g/dL), moderate (Hb: 10–11 g/dL), and mild anemia in different types of anemia in the PolSenior study.

	Severe Anemia % (*n*)	Moderate Anemia % (*n*)	Mild Anemia % (*n*)
**Gender**			
Males ^a^	52.0 (26)	44.2 (34)	68.9 (314)
Females	48.0 (24)	55.8 (43)	31.1 (142)
**Age Cohorts**			
65–69	10.0 (5)	2.6 (2)	5.3 (24)
70–74	8.0 (4)	6.5 (5)	12.7 (58)
75–79	10.0 (5)	18.2 (14)	11.8 (54)
80–84 ^a^	6.0 (3)	13.0 (10)	17.5 (80)
85–89	30.0 (15)	26.0 (20)	24.6 (112)
90+	36.0 (18)	33.8 (26)	28.1 (128)
**MCV**			
Normocytosis ^a^	58.0 (29)	76.6 (59)	92.8 (423)
Microcytosis ^a^	38.0 (19)	13.0 (10)	2.6 (12)
Macrocytosis	4.0 (2)	10.4 (8)	4.6 (21)
**Type of Anemia (Classified According to ROC Curve Analysis)**			
**Anemia with Nutritional Deficiency**			
Folate only	4.0 (2)	6.5 (5)	8.1 (37)
B_12_ only	10.0 (5)	18.2 (14)	21.9 (100)
IDA only ^a^	26.0 (13)	15.6 (12)	10.7 (49)
Folate + B_12_	0.0 (0)	1.3 (1)	3.7 (17)
IDA + folate/B_12_^a^	38.0 (19)	26.0 (20)	7.0 (32)
All deficiency ^a^	78.0 (39)	67.5 (52)	51.5 (235)
**Anemia without Nutritional Deficiency**			
CKD only	12.6 (16)	15.6 (12)	9.4 (43)
AI	3.9 (5)	2.6 (2)	7.7 (35)
CKD + AI	4.7 (6)	5.2 (4)	4.8 (22)
Unknown ^a^	7.1 (9)	9.1 (7)	26.5 (121)
All non-deficiency ^a^	28.3 (36)	32.5 (25)	48.5 (221)
**Total**	100.0 (50)	100.0 (77)	100.0 (456)

^a^ *p* < 0.05 for mild vs. moderate and severe comparison; AI—anemia of inflammation; CKD—chronic kidney disease; IDA—iron deficiency anemia; ROC—receiver operating characteristic.

**Table 6:** Criteria of nutritional deficiency and anemia of inflammation in NHANES III study, “Health and Anemia” study, and PolSenior study (in the latter criteria established according to three different formulas).

Study	Iron Deficiency	Vitamin B_12_ Deficiency	Folate Deficiency	Anemia of Inflammation
NHANES III	2 or 3 of following: TfS < 15% serum ferritin < 12 ng/mL EP >1.24 µ mol/L	serum B_12_ < 200 pg/mL	RBC folate 102.6 ng/dL or serum folate 2.6 ng/mL	serum iron < 60 μg/dL without evidence of iron deficiency
Health and Anemia	serum iron < 50 μg/dL in women and <60 μg/dL in men serum ferritin < 15 ng/mL TfS < 16% or TIBC > 450 μg/dL	serum B_12_ < 200 pg/mL and MCV > 95 fL	serum folate < 3.0 pg/mL and MCV > 95 fL	serum ferritin > 100 ng/mL TfS 25–50% TIBC < 250 μg/dL
PolSenior (WHO criteria)	serum ferritin < 15 ng/mL	serum B_12_ < 200 pg/mL	serum folate < 3.0 pg/mL	serum iron < 60 μg/dL and sF ≥ 15 ng/mL
PolSenior (lowest 2.5 percentile)	serum ferritin < 25 ng/mL	serum B_12_ < 75 pg/mL	serum B_12_ < 1.6 pg/mL	serum iron < 35.1 μg/dL and sF ≥ 25 ng/mL
PolSenior (ROC analysis)	serum ferritin < 58 ng/mL	serum B_12_ < 186 pg/mL	serum B_12_ < 2.4 pg/mL	serum iron < 78 μg/dL and serum ferritin ≥ 78 ng/mL

EP—erythrocyte protoporphyrin; ROC—receiver operating characteristic; TfS—transferrin saturation; TIBC—total iron-binding capacity; WHO—World Health Organization.

## Data Availability

The data presented in this study are available on request from the corresponding author. The data are not publicly available because the above study is part of a larger project. The participants of the project agreed to present the data only in aggregate form. Access to individual data requires the consent of the Scientific Committee of the PolSenior project.
